# Establishing and evaluation of a polymerase chain reaction for the detection of *Echinococcus multilocularis* in human tissue

**DOI:** 10.1371/journal.pntd.0009155

**Published:** 2021-02-25

**Authors:** Johannes Grimm, Julian Krickl, Annika Beck, Juliane Nell, Monika Bergmann, Dennis Tappe, Beate Grüner, Thomas FE Barth, Klaus Brehm

**Affiliations:** 1 Institute of Pathology, University of Ulm, Ulm, Germany; 2 Consultant Laboratory for Echinococcosis, Institute for Hygiene and Microbiology, University of Wuerzburg, Wuerzburg, Germany; 3 Division of Infectious Diseases, University Hospital and Medical Center, Ulm, Germany; Universidade Federal de Minas Gerais, BRAZIL

## Abstract

**Background:**

Alveolar echinococcosis (AE) is caused by metacestode larva of the tapeworm *Echinococcus multilocularis*. AE diagnostics currently rely on imaging techniques supported by serology, but unequivocal detection of AE is difficult. Although polymerase chain reaction (PCR)-based methods to detect tapeworm DNA in biopsies have been suggested for several species, no validated protocol adhering to accepted guidelines has so far been presented for AE diagnostics. We herein established a PCR protocol for metacestode biopsies and technically evaluated the method using isolated parasite DNA and cells, biopsies of clinically relevant material, and formalin fixed paraffin-embedded (FFPE) human tissue blocks. We compared the results with an immunochemical (IHC) approach using the monoclonal antibody Em2G11 specific for the antigen *Em2* of *E*. *mulitlocularis*.

**Methodology/Principal findings:**

Based on tapeworm 12S rDNA sequences we established and validated a PCR protocol for robust detection of as little as 50 parasite cells per specimen and report 127 cases of positive identification of *Echinococcus* species in samples from humans and animals. For further validation, we analyzed 45 liver, heart, brain, and soft tissue samples as well as cytological probes of aspirates of FFPE-material from 18 patients with clinically confirmed AE. Of each patient we analyzed (i) fully viable lesions with laminated layer; (ii) tissue with mAbEm2G11-positive small particles of *E*. *multilocularis* (*spems*); (iii) mAbEm2G11-negative tissue adjacent to the main lesion; and (iv) lymph node tissue with mAbEm2G11-positive *spems*. To identify the areas for the PCR-based approach, we performed IHC-staining with the monoclonal antibody *Em2G11*. Micro-dissected tissue of these areas was then used for PCR-analysis. 9 of 15 analyzed samples with viable *E*. *multilocularis* lesions with laminated layer were positive by PCR. Of this group, all samples preserved for less than 6 years (6/6) were tested positive. 11 of 15 samples of *spems* and 7 of 9 samples of the control group mAbEm2G11-negative tissue were negative by PCR. We further show that all probes from lymph nodes with *spems* are PCR negative.

**Conclusions/Significance:**

We present a sensitive PCR method for the detection of *E*. *multilocularis* in human tissue, particularly in fresh biopsy material and tissue blocks stored for less than 5 years. While the diagnostic sensitivity of material containing only *spems* was higher using IHC, PCR detection was possible in IHC negative liver tissue and in patients with negative serology. Our results support the view that *spems* do not contain parasitic DNA or viable cells of the parasite. *spems* thus most probably do not directly contribute to metastasis formation during AE.

## Author summary

Alveolar echinococcosis (AE) is a potentially lethal zoonosis during which metacestode larval tissue of the tapeworm *Echinococcus multilocularis* grows like a malignant tumor, infiltrating the human liver. Current AE diagnosis, which mainly relies on imaging techniques (ultrasound, computed tomography, magnetic resonance tomography) supported by serology, is not always suitable to unequivocally identify an infection. In this work the authors present a PCR-based approach to detect the parasite in biopsy material taken from patients. The method was first validated for routine laboratory use employing isolated parasite DNA as well as defined numbers of parasite cells in clinically relevant settings. The method was then verified under clinically relevant settings using pathology samples from patients with defined AE. Particularly in cases when these samples had been stored for less than 6 years, the PCR methodology was highly suitable to identify the infection, even when serology was negative. The authors show that small particles of *E*. *multilocularis* (*spems*), containing surface material of the parasite and often located in the draining adjacent lymph nodes, are PCR negative and, thus, most probably do not contain viable parasite cells. The authors conclude that in cases of doubtful serology and imaging, a combination of immunochemical staining methods with PCR are reliable means to unequivocally detect the infection.

## Introduction

The metacestode larval stage of the fox-tapeworm *Echinococcus multilocularis* is the causative agent of alveolar echinococcosis (AE), a potentially lethal zoonosis prevalent in the Northern Hemisphere [1, 2]. Infections of intermediate hosts (rodents, humans) are initiated through oral uptake of tapeworm eggs which contain the embryonic oncosphere larval stage. After hatching in the host intestine, the oncosphere penetrates the intestinal epithelium and gains access to the inner organs where it undergoes a metamorphotic transition towards the metacestode stage [3]. The metacestode consists of posteriorized larval tissue which grows infiltratively, like a malignant tumor, into the surrounding host tissue [4, 5]. Larval proliferation and growth is decisively driven by a population of pluripotent parasite stem cells (the ‘germinative cells’) which give rise to all differentiated cells (e.g. muscle cells, nerve cells, storage cells, tegument) of the metacestode that make up the cellular germinal layer (GL) [4, 6]. The GL is surrounded by an acellular ‘laminated layer’ (LL) which consists of a mesh of highly glycosylated mucins, supposed to be produced and shed off the tegumental cells [7]. The LL has a crucial role in protecting the parasite from the immune system of the host [8] and, in *E*. *multilocularis*, reaches about 10–12 μm in thickness [9]. The life cycle of the closely related dog-tapeworm *E*. *granulosus*, the causative agent of cystic echinococcosis (CE), is very similar to that of *E*. *multilocularis* with some noticeable differences in the host spectrum (mostly domesticated species) and larval morphology [3]. In particular, the LL of *E*. *granulosus* is more complex and thicker than that of *E*. *multilocularis* (several mm) [9] and, probably as a consequence of this, the *E*. *granulosus* metacestode grows as large, fluid-filled cysts instead of infiltrative tissue. Larval metacestode development of *E*. *multilocularis* almost always occurs first within the liver of the intermediate host but, in prolonged infections, may lead to metastasis formation in other organs by yet unknown mechanisms [2]. The *E*. *granulosus* metacestode, on the other hand, has a less pronounced organs-tropism towards the host liver and also affects other organs such as the lung or the brain [2]. Furthermore, it is thought that *E*. *granulosus* tissue does not lead to metastases. While CE is more amenable to surgical and chemotherapeutic intervention, AE treatment is still challenging [10]. In only about 20% of all cases, surgical removal of the parasite tissue is possible and still remains the only option for cure of the disease. In the remaining cases, chemotherapy using benzimidazoles (BZ) is the current treatment of choice. However, BZ are associated with severe adverse side effects and *in vivo* only have parasitostatic activity against AE. BZ chemotherapy thus has to be given for prolonged periods of time (often life-long) [11].

Infections due to larval cestodes, in particular cysticercosis and echinococcosis, are listed by the World Health Organization (WHO) among the Neglected Tropical Diseases (NTDs) and account for approximately 2.5 million DALYs (disability adjusted life years lost) annually [12]. Regarding impact on human health, larval cestodes thus rank second, after schistosomiasis, among all helminth-borne NTDs [13, 14]. Of all diseases caused by tapeworms of the genus *Echinococcus*, AE and CE are the most important human infections world-wide and, according to the World Health Organization, account for 1–3 million DALYs annually [14, 15]. In both cases of the disease, diagnostics mainly relies on imaging techniques such as ultrasonography or computer tomography, which identify the larval parasite tissue in host organs, supported by serology [16]. A number of immunodiagnostic tests for echinococcosis detection have already been developed and rely on either crude antigens derived from protoscoleces or metacestode tissue, on recombinant antigens, or on purified LL fractions, with different sensitivities and specificities [16]. In general, although existing serological tests are mostly reliable in detecting active infections, immunodiagnostics is rather poor in differentiating between AE and CE due to the high genetic similarity between *E*. *multilocularis* and *E*. *granulosus* [17]. Furthermore, particularly in cases of unusual locations of parasite lesions, serological tests frequently fail in identifying the disease [16]. Histology supplemented by immunochemical detection of parasite tissue and, in particular, the laminated layer as a hallmark of the genus *Echinococcus*, is thus a decisive complementary technique to imaging and serology [2, 10, 18, 19]. Furthermore, molecular diagnostic tools such as PCR are increasingly used for unequivocal parasite detection from biopsy material [2]. During recent years, several PCR protocols have thus been presented by which tissue of *E*. *multilocularis*, *E*. *granulosus* or other taeniid cestodes has successfully been detected in native or in formalin-fixed paraffin-embedded tissue samples [20–22]. However, in none of these cases the PCR procedures have been assessed according to generally accepted guidelines [23–26] for the validation of diagnostic nucleic acid amplification tests (including procedures that determine sensitivity of the test in clinically relevant settings). In the present work we introduce a PCR amplification protocol for the reliable detection of *Echinococcus* parasite lesions in biopsy material and paraffin blocks. This protocol was tested for sensitivity using isolated parasite DNA as well as defined numbers of *Echinococcus* cells in clinically relevant settings.

The established PCR protocol was used for the detection of parasite tissue in a variety of patient samples and for a closer examination of the molecular nature of the previously described *spems* (small particles of *E*. *multilocularis*) [18]. *spems* have originally been found in *E*. *multilocularis* patients to be located outside the main lesions in necrotic tissue, liver sinusoids, and lymphatic tissue and can be stained using the monoclonal antibody Em2G11 specific for the parasite’s LL [27]. Furthermore, it has been shown that BZ treatment significantly increases the amount of *spems* in human hepatic AE lesions [28]. Our results indicate that *spems* do not contain viable parasite cells but are rather a product of shedding of the LL during parasite growth.

## Material and methods

### Ethics statement

The study is in accordance with the guidelines for the use of archived human tissue as outlined by the *Zentrale Ethikkommission bei der Bundesärztekammer* [29]. All experiments involving animals were carried out in accordance with European and German regulations on the protection of animals (*Tierschutzgesetz*). Ethical approval of the study was obtained from the local ethics committee of the government of Lower Franconia (permit no. 55.2 DMS 2532-2-354).

### Organisms and culture methods

All experiments from PCR evaluation were carried out using the natural *E*. *multilocularis* isolate H95 [17] which was maintained by serial intraperitoneal passages in *Meriones unguiculatus* (Mongolian jird) as previously described [30]. For protoscolex isolation the recent isolate GH09 was used, which was obtained from accidental infections of Old World Monkeys in a breeding enclosure [31]. Metacestode *in vitro* cultivation and the isolation of parasite primary cells were carried out using isolate H95 essentially as described previously [32]. For *E*. *multilocularis* cell counting, samples (1:200 dilution in PBS) of freshly isolated primary cells were stained with Hoechst 33342 (Invitrogen) and counted using a Neubauer Chamber and a fluorescence microscope (Company). After cell counting the required cell numbers were diluted in PBS and mixed with either freshly isolated liver tissue of *M*. *unguiculatus* or with defined cell numbers of the human liver cancer cell line Hep G2.

### Nucleic acid extraction

For nucleic acid isolation from protoscoleces or parasite cell material (mixed with either human cells or *M*. *unguiculatus* tissue) the ‘QIAamp DNA Mini’ kit (Qiagen) was used according to the manufacturer’s protocol for ‘DNA isolation from tissue’. Briefly, parasite/host mixture samples were mixed with glass beads and vigorously shaken for 10 min in a 1.5 ml reaction tube. Then, 180 μl ATL-buffer (Qiagen) and 20 μl proteinase K solution (20 mg/ml) were added and probes were incubated for 3 h at 56°C. After addition of 200 μl of buffer AL and 15 sec of vigorous shaking the solution was added to a QIAamp spin column and centrifuged for 1 min at 6000 x g (sample binding). After transferring the column into a new tube, 500 μl buffer AW1 was added and, again, the column was centrifuged for 1 min at 6000 x g (first washing step). After adding the column to a fresh tube, 500 μl buffer AW2 were added and the column was centrifuged for 3 min at 20000 x g (second washing step). The column was finally transferred to a new tube and 200 μl buffer AE were added. After 5 min incubation at room temperature, tube and column were centrifuged for 1 min at 6000 x g (elution) and the flow-through stored at -20°C till use in PCR. For analyses on the detection limit of the assay using isolated parasite cells, 1 μl of the flow-through were used for PCR. In the case of human biopsy samples, 10 μl of the flow-through are routinely used.

For paraffin-block samples, microdisected material of microscopically marked areas on tissue slides was incubated in 1 ml n-octan for 1 h under light shaking. The sample was centrifuged for 10 min at 20000 x g, the pellet was dissolved in 1 ml ethanol and, again, centrifuged for 10 min at 20000 x g. After removing the ethanol supernatant and drying of the sample at 56°C for 1 h, buffer ATL was applied and the sample was further processed as described above using the QIAamp DNA mini kit.

### Consultant laboratory analyses on patient samples

At the consultant laboratory for echinococcosis (University of Würzburg), serological and molecular biological analyses on material of patients with suspect of echinococcosis of all areas of Germany are routinely carried out. Incorporated in this study were all samples (n = 417) that had been submitted for PCR analysis between September 2011 and June 2020. Of these samples, 389 concerned fresh biopsy material (usually fine needle biopsies) that had been sent either in ethanol or without treatment within one week of isolation. Twenty-five milligrams of patient material was subsequently used for DNA extraction (final eluate 200 μl) using the QIAamp DNA mini kit (Qiagen) essentially according to the manufacturer’s instructions. Ten milliliters of the eluate was then analyzed by PCR using primer-set 1 and PCR protocol A (see below).

In 28 cases, paraffin embedded material not older than 6 months had been analyzed. To this end, the surface of the paraffin block was scratched using a scalpel, the gathered material was incubated in 1 ml n-octan, vigorously shaken for 1 h, and then centrifuged for 10 min at 20.000 g. The pellet was dissolved in 70% ethanol, centrifuged again at 20.000 g. The air-dried pellet was subsequently used for DNA extraction using the QIAamp DNA mini kit as described above. In all cases, PCR products were subsequently analyzed by capillary electrophoresis using the QIAxcel Advanced system (Qiagen) and sequenced by Sanger sequencing (Microsynth Seqlab, Göttingen, Germany) using one of the PCR primers used for amplification. Species differentiation was performed on the basis of BLASTN comparisons of the obtained sequences with nucleotide sequences deposited at the ncbi database (https://www.ncbi.nlm.nih.gov/nucleotide). Criteria for positive identification of a cestode species were at least 99% identity to the respective sequence deposited in the *ncbi* database. During the entire period of investigation (2011–2020) the technical protocols for DNA extraction and PCR analysis were not changed.

### Evaluation of PCR on selected patient and tissue samples

Formalin-fixed paraffin-embedded (FFPE) tissue of 22 different patients was retrieved from the archives of the Institute of Pathology, University of Ulm. The samples were anonymized according to German law for correct usage of archival tissue for clinical research. Eighteen of these patients had clinical and immunochemically confirmed infection with *E*. *multilocularis*. These samples encompassed tissues of 9 female patients and 8 male patients; for one patient, the gender was not known. The mean age of this cohort was 45 years. The tissue cohort included: liver (n = 29), brain (n = 2), heart (n = 1), mamma (n = 1), soft tissue (n = 3), lymph node (n = 6). Furthermore, three cytological aspirate samples were analyzed. In 13 cases, serological data of the patients were known (S1 Table).

To test the efficiency of the approach, the samples were divided in different groups including various areas of the *Echinococcus* lesion. The first group (n = 15) included material of the viable lesion with fragments of the LL in a necrotic background (group 1). The second group (n = 15) contained samples of mAbEm2G11-positive *spems* in the neighborhood of the main lesion (n = 13) or cytological material (n = 2) gained by aspiration (group 2). mAbEm2G11 negative tissue adjacent to the main lesion (n = 9) formed the third group (group 3). The next group included six lymph nodes which contained mAbEm2G11-positive *spems* in the germinal center of the lymph follicle (group 4).

The control group consisted of liver tissue samples of two patients with non-infected lymph node tissue (n = 2). An assignment of the samples to the different groups is shown in S1 Table.

### Staining

Hematoxylin and eosin (H.E.) staining and immunochemistry was performed according to standard protocols. In brief, the resected tissue was fixed in 4% buffered formaldehyde for at least 36 hours. Serial sections of about 3 μm from paraffin blocks with representative tissue were performed with a microtome. Paraffin was dissolved with xylol and a descending alcohol series. For antigen retrieval in immunohistochemistry, the slides were treated with citrate buffer pH 6 for 20 minutes in a pressure cooker. As primary antibody, the monoclonal antibody Em2G11 (IgG_1_) was used. This antibody is specific for the *E*. *multilocularis* antigen Em2 [15, 24]. The antibody was diluted in Antibody Diluent (Zytomed Systems, Berlin; 1:100). The slides were incubated with 100μl of primary antibody in a humid chamber at room temperature for 30 minutes. As detection system, the DAKO REAL Detection System, Alkaline Phosphatase/Red (DAKO, Carpintera, CA, USA) was used according to the manufacturer’s protocols. The tissue was stained with hematoxylin after immunochemistry. Staining performed without primary antibody was used as negative control.

### Tissue preparation

In a pilot experiment, the minimum amount required for a positive PCR result of *E*. *multilocularis* infected tissue was tested. Areas on tissue sections (slide thick 5 μm) of *E*. *multilocularis* liver lesions with different diameters (1mm, 2mm, 5mm, 10mm) were analyzed. These areas were selected under microscopic control, the area of interest was micro-dissected, and the tissue treated with proteinase K as described below. All samples with a diameter larger than 1 mm were tested positive by PCR. Based on this analysis we used micro-dissection of tissue areas that had at least a diameter of 2 mm and a slide thickness of 5 μm. The immunohistochemical stainings with mAbEm2G11 were analyzed at a microscope and the different areas for each group were marked. After dissolving of paraffin with xylol and a descending alcohol series, the unstained material of marked tissue areas was micro-dissected and transferred into in a reaction tube containing a mix of proteinase K (20 mg/ml) and TEN-buffer (1 mM EDTA, 10 mM TRIS-HCl, 0,1 M NaCl, pH 8,0). The ratio of proteinase K and TEN-buffer was 1 to 11. The tubes containing this mix were incubated in a thermomixer at 62°C overnight. The reaction was stopped for 5 minutes at 95°C.

### Serological analyses

For serological analysis of all patients listed in S1 Table, we used enzyme linked immunosorbent assays (*E*. *multilocularis*–ELISA Em2plus, Nr. 9300, Bordier Affinity, Crissier, Switzerland or SERION ELISA classic *Echinococcus* IgG, virion/serion, Wuerzburg, Germany). The tests were performed and evaluated according to the manufacturers protocol. Serological testing for all remaining patients (biopsy and formalin-fixed material at the consultant laboratory for echinococcosis) was performed using an in house enzyme linked immunosorbent (ELISA) test using *E*. *multilocularis* crude antigen essentially as previously described [33].

### PCR conditions

For PCR evaluation, two sets of primers were used. Primer-set 1 had originally been established for phylogenetic investigations on cestodes [34], and already been used for PCR detection of *Echinococcus* in biopsies [35] and consisted of primers 12S-60F (5’-TTA AGA TAT ATG TGG TAC AGG ATT AGA TAC CC—3’) and 12S-375R (5’-AAC CGA GGG TGA CGG GCG GTG TGT ACC-3’). Primer-set 2 was designed to bind more specifically to *Echinococcus* 12S rDNA and consisted of primers 12S-newF (5’-CAC AGT GCC AGC ATC TGC GG-3’) and 12S-newR (5’-AAC CGA GGG TGA CGG GCG GTG TG TAC-3’). For primer-set 1 we used a previously established PCR protocol as described [35] with the exception that we added another initial denaturation step of 10 min at 95°C. The final protocol contained the following steps: two initial denaturation steps of 10 min at 95°C and 15 min. at 93°C, followed by 49 cycles of 11 sec at 93°C, 27 sec at 55°C, and 71 sec at 73°C. Final elongation was carried out for 5 min at 72°C. The expected amplicon size for these primers is 374 bp. This protocol is referred to as PCR protocol A throughout the manuscript. For primer-set 2 we used PCR protocol B with an initial denaturation of 1 min at 95°C followed by either 30 (PCR protocol B30) or 49 (PCR protocol B49) cycles of 30 sec at 95°C, 30 sec at 63°C, and 30 sec at 68°C. Final elongation was carried out for 5 min at 72°C. The expected amplicon size of for these primers was 493 bp. For PCR amplification of a short amplicon on selected FFPE samples, primers 12S-newF (see above) and 12S-newR2 (5’-GAA TCA CTC ACT GCC AAA CCA-3’) were used (expected amplicon size 216 bp) in a PCR protocol (protocol C) with 1 min at 95°C, 49 cycles of 30 sec at 95°C, 30 sec at 55°C or 63°C (as indicated), and 30 sec at 72°C. Terminal elongation of protocol C was 5 min at 72°C. In analyses on detection method sensitivity, 1 μl DNA solution of the extraction procedure was analyzed in a 50 μl reaction using the AmpliTaq Gold–kit (Thermo Scientific, Germany) according to the manufacturer’s instructions. In the case of paraffin-embedded material or microscope slides, 10 μl of the extraction solution was used employing the AmpliTaq kit. All PCRs were run on a Biometra Trio analyzer (Jena Analytik, Germany), PCR products were subsequently visualized after capillary electrophoresis by the QIAxcel Advanced system (Qiagen) essentially according to the manufacturer’s instructions, or on 1.5% agarose gels. PCR products were directly sequenced by Sanger sequencing (Microsynth Seqlab, Göttingen, Germany) using one of the PCR primers used for amplification. Sequences were then analyzed by BLASTN.

## Results

### PCR technical validation

**Fig 1:**
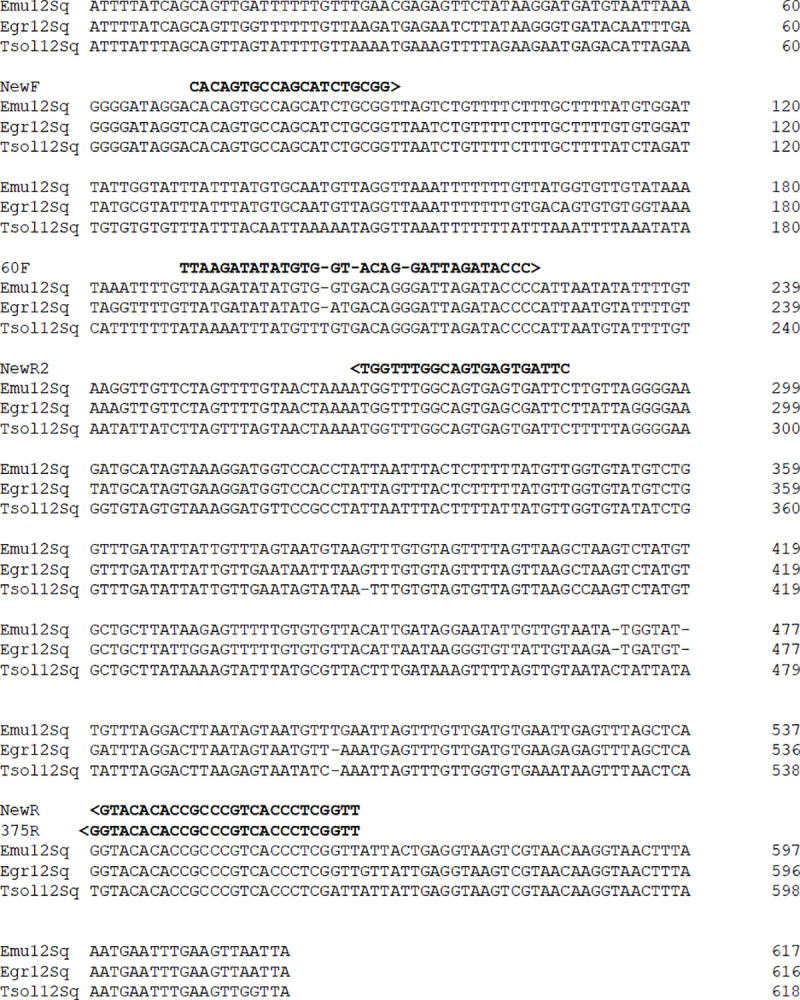
DNA sequence comparison of taeniid cestode 12 rDNA regions. Displayed is a CLUSTALW alignment of 12 S rDNA sequences deriving from *E*. *multilocularis* (Emu12Sq; GenBank accession no. AB018440.2), *E*. *granulosus* (Egr12Sq; NC_044548.1), and *Taenia solium* (Tsol12Sq; AB086256.1). Dashes have been included to maximize the alignment. DNA sequences of primers NewF and 60F used in this study are shown above the alignment in 5‘-3‘-orientation. The sequences of primers NewR and 375R are displayed in reverse orientation.

For establishing a validated PCR method to detect *Echinococcus* DNA in biopsies we concentrated on a previously established protocol that had successfully been used to detect *E*. *multilocularis* and *E*. *granulosus* infections in nine patients in Poland [35]. This method was of limited use for routine laboratory diagnostics since it had exclusively been established for biopsies (not paraffin-block tissue or microscope slide material) and had never been validated concerning sensitivity and specificity under clinically relevant settings according to established guidelines [23–25]. As shown in Fig 1, the primers used by Myjak et al. [35] (Primer-set 1 in the present study) did not perfectly match the published mitochondrial 12S rDNA sequences of *E*. *multilocularis*, *E*. *granulosus*, and the related taeniid tapeworm species *Taenia solium*. While the reverse primer 375R displayed 100% similarity to 12S rDNA sequences of *E*. *multilocularis* and *E*. *granulosus*, there was one mismatch at the 3’ end of the primer when compared to the *T*. *solium* sequence. Forward primer 60F displayed several mismatches in both *Echinococcus* species as well as in *T*. *solium* (Fig 1). We thus considered primer-set 1 not perfectly suited for PCR amplification of *Echinococcus* DNA from patient samples and designed another pair of primers (primer-set 2 in the present study), which displayed high similarity to 12S rDNA of all three species (Fig 1). Since primer-set 1 displayed a considerable difference in primer melting temperatures (55°C for 12S-60 F and 69°C for 12S-375R) we considered primer-set 2 more suitable since the two primers only had a difference of 6°C in melting temperatures. For PCR conditions we chose the original program as designed by Myjak et al. [35] for primer-set 1 (PCR protocol A) and we used a standard program with either 30 or 49 cycles for primer-set 2 (PCR protocol B).

In a first set of experiments we tested all primer-sets and PCR conditions on *E*. *multilocularis* DNA isolated from protoscoleces (100 pg/μl concentration) and on DNA samples isolated from parasite liver lesions of an infected murine host (*M*. *unguiculatus*). In these assays, Primer-set 1 produced clear positive bands for parasite DNA and for the intermediate host sample (S1 Fig). Primer-set 2 also produced positive bands for all these conditions when PCR protocol B49 (49 cycles) was used, whereas primer-set 2 with PCR protocol B30 (30 cycles) only yielded positive results for isolated parasite DNA (S1 Fig). In all following experiments were therefore employed PCR protocol B49 for primer-set 2.

Next, we produced serial, 10-fold dilutions from isolated parasite DNA (from 10^4^ pg/μl to 10^−3^ pg/μl) and tested these using primer-set 1 (PCR protocol A) and primer-set 2 (PCR protocol B49). In both cases we obtained clear positive results to a dilution of 1 pg/μl, whereas 0.1 pg/μl only produced faint bands and no bands were obtained at or below 10^−2^ pg/μl. To further verify these results, we produced five independent isolations of protoscolex DNA and tested each at concentrations of 100, 10, 1, and 0.1 pg/μl. As shown in Table 1, both primer-set 1 and primer-set 2 yielded positive results in 5 of 5 samples for concentrations of 1 pg/μl and higher. For 0.1 pg/μl we obtained 4/5 positive results for primer-set 1 and only 1/5 positive samples for primer-set 2. These data indicate a somewhat higher level of sensitivity of primer-set 1 at lower concentrations of parasite DNA, which could in part be due to the lower (less stringent) annealing temperature for primer-set 1 (55°C) when compared to primer-set 2 (63°C).

**Table 1:** PCR-Detection of *E*. *multilocularis* 12 rDNA in serial dilutions of isolated chromosomal DNA.

Conc. pg/μl	100	10	1	0,1	0
Primerset 1*	5/5	5/5	5/5	4/5	0/5
Primerset 2*	5/5	5/5	5/5	1/5	0/5

* shown are positive results out of five independent experiments.

Next, we tested effects of the intermediate host matrix on PCR detection. To this end, we produced ten-fold, serial dilutions of isolated parasite primary cells, which mainly consist of germinative stem cells [6], from 10^6^ to 10^2^ cells, mixed these with 25 mg freshly isolated liver tissue of *M*. *unguiculatus*, applied the DNA isolation standard protocol, and carried out PCR. As shown in Table 2, in nine independent experiments primer-set 1 yielded positive results for all cases using cell numbers of 10^4^ or higher. For 10^3^ cells, only 6 of 9 samples were positive and for 10^2^ cells, only 2 of nine were positive using primer-set 1. Again, primer-set 2 was less sensitive than primer-set 1, particularly at cell numbers below 10^4^ (Table 2). Since a 1:200 dilution of the originally isolated DNA was used for PCR amplification, these results indicated that primer-set 1 can successfully amplify samples with as few as 50 parasite cells whereas primer-set 2 required 500 parasite cells for unequivocal detection.

**Table 2:** PCR-Detection of *E*. *multilocularis* 12 rDNA on isolated parasite cells.

Cell number	10^6^	10^5^	10^4^	10^3^	10^2^
Primerset 1*	9/9	9/9	9/9	6/9	2/9
Primerset 2*	9/9	9/9	5/9	3/9	3/9

* shown are positive results out of nine independent experiments.

**Fig 2:**
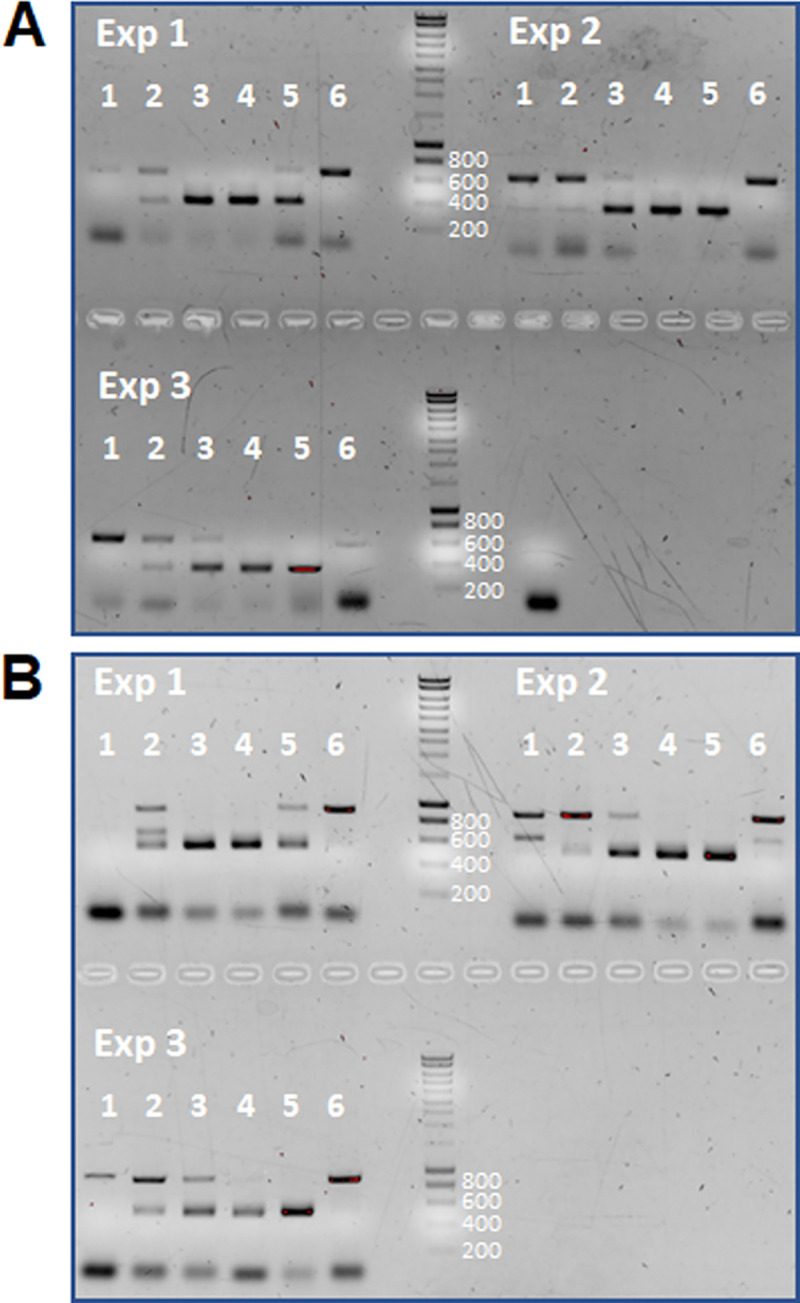
PCR analysis of mixtures of parasite cells and human HepG2 cells. A constant number of 10^6^ human HepG2 cells has been mixed with 10^2^ (lanes 1), 10^3^ (lanes 2), 10^4^ (lanes 3), 10^5^ (lanes 4), 10^6^ (lanes 5) and no (lanes 6) parasite cells prior to DNA extraction and PCR. Analyses in (A) have been performed with primer-set 1 (expected size 374 bp), those in (B) with primer-set 2 (expected size 493 bp). Exp1-3 indicates the results of three independently performed experiments. PCR amplificates have been separated on a 1.5% agarose gel and stained with ethidium bromide. DNA marker sizes (in bp) are indicated to the right of the marker lane.

Since the normal setting for PCR diagnosis of AE involves human tissue, we also tested the effect of human cells on the detection of parasite cells. To this end, a constant number of 10^6^ HepG2 human liver carcinoma cells was mixed with serial, 10-fold dilutions of parasite cells (10^6^ to 10^2^). As shown in Fig 2, a number of 10^4^ parasite cells analyzed by primer-set 1 always yielded clear positive results (374 bp expected size for amplicon of parasite 12S r-DNA) in three independent experiments, whereas at a cell number of 10^3^, mixed products between the parasite 12S r-DNA band and an unspecific amplification product of around 700 bp were obtained. Upon sequencing, the non-specific product was shown to derive from human 12S r-DNA. At a concentration of 10^2^ parasite cells, only the host product was amplified (Fig 2). In the case of primer-set 2 we obtained similar results as for primer-set 1 (Fig 2). However, at least in two samples mixed products were also obtained for 10^4^ parasite cells and at cell number below 10^4^, host contaminating products were more intense that the parasite-specific 12 rDNA band. These data again indicated a higher sensitivity of primer-set 1 for PCR detection of *E*. *multilocularis* DNA and that an equivalent of only 50 parasite cells can be detected in mixture with host cells.

To obtain an estimation of how much parasite material (e.g. in biopsies) would be necessary to account for 50 or 500 cells, we carried out fluoresce microscopy on *in vitro* cultivated *E*. *multilcularis* metacestode vesicles. Vesicles after 3 weeks of cultivation *in vitro* were fixed and stained with propidium iodide to visualize the nuclei. We then carried out a Z-stack analysis and counted whole nuclei numbers per parasite tissue volume (S2 Fig). According to these analyses, *in vitro* cultivated metacestode tissue contained 0,00184 nuclei per μm^3^, which sums up to 1840 nuclei in a tissue block of 100 per 100 per 100 μm, which can easily be taken up even by fine needle biopsy.

**Fig 3:**
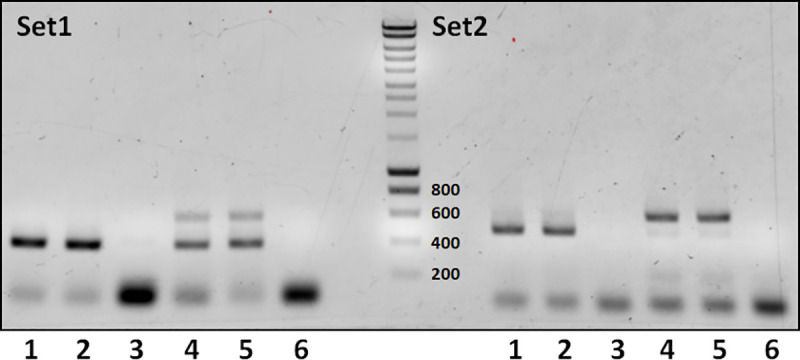
PCR analysis of samples taken directly from parasite lesions and in a distance of 1 cm to lesions. In lanes 1 and 2, samples had been taken directly from liver lesions, in samples 4 and 5 in 1 cm distance. Lanes 3 and 6 are negative controls. M indicates DNA marker lane. Set1 (left panel) has been performed with primer-set 1 (expected band size 374 bp), Set2 (right panel) was performed with primer-set 2 (expected band size 493 bp). PCR products have been separated on a 1.5% agarose gel and stained with ethidium bromide. DNA marker sizes (in bp) are indicated to the right of the marker lane.

Finally, we tested on parasite material isolated from the natural intermediate host (*M*. *unguiculatus*) whether parasite DNA can only be detected when taken directly from parasite lesions or whether liver tissue that surrounds parasite lesions also contains parasite DNA. To this end, we independently isolated samples of metacestode tissue from an infected jird and we also took samples of liver tissue in a distance of 1 cm to parasite lesions. As expected, samples taken directly from metacestode tissue gave clear positive results for primer-set 1 and primer-set 2 (Fig 3), whereas in the case of primer-set 1 mixed products were obtained for samples taken in a 1 cm distance. Cloning and sequencing indicated that these were products of both parasite 12S rDNA and *Meriones* 12S-rDNA. In the case of primer-set 2, only *Meriones* 12S rDNA was amplified from liver tissue in a distance of 1 cm from lesions (Fig 3). These data indicated that parasite DNA concentration in 1 cm distance of active lesions is already very low and either yields no or doubtful results.

### PCR analysis of biopsy material from humans and animals

At the consultant laboratory for echinococcosis (University of Würzburg), we carried out a total number of 417 PCR analyses on fresh biopsies (n = 389) and paraffin embedded material (n = 28), using primer-set 1 and PCR protocol A (Table 3). Of these 417 analyses, 128 yielded a clear positive result in PCR (102 for fresh material, 26 for paraffin embedded material) and also allowed species identification by DNA sequencing. When using fresh biopsy material from different human organs we obtained positive results for *E*. *multilocularis* in 57 cases, for *E*. *granulosus sensu stricto* in 34 cases, and even one case of *E*. *ortleppi* (formerly known as the *E*. *granulosus* strain G5), and one case of *Taenia martis* (Table 3). As expected, most of the positive cases for *E*. *multilocularis* and *E*. *granulosus* were obtained for biopsy material from liver tissue or cysts, followed by lung biopsies, but we also positively detected the parasites in samples taken from lymph node, muscle, spleen, and sputum as well as from material in peritoneal locations (Table 3). Furthermore, we positively identified *E*. *multilocularis* in liver samples taken from monkeys, domestic dogs, wild boar, and red deer, and we identified an *E*. *equinus* (formerly known as *E*. *granulosus* strain G4) infection in a horse from Germany (Table 3). Finally, we could also clearly identify *E*. *multilocularis* and *E*. *granulosus* infections on material recently embedded (not older than 6 months) in paraffin blocks (Table 3). The respective samples mostly derived from liver but also from brain, spleen, abdomen, and lung (Table 3). Taken together, these data indicated that primer-set 1 can efficiently be used to detect not only a variety of *Echinococcus* species, but also other teaniid cestodes in biopsies and paraffin embedded material from a broad range of hosts.

**Table 3:** PCR positive diagnosis of different *Echinococcus* species in human and animal biopsy material.

Material analyzed species detected	human biopsy	paraffin block*	monkey^§^	*Sus scrofa*	*Equus caballus*	*Cervus elaphus*	dog
*E*. *multilocularis*	liver (41) lung (3) lymph node (2) muscle (4) spleen (1) peritoneum (3) sputum (1) ascites (1) drainage (1)	liver (14) brain (1) spleen (1) abdomen (2)	liver (3)	liver (1)		liver (1)	liver (3)
*E*. *granulosus*	liver (27) lung (5) sputum (1) peritoneum (1)	liver (4) peritoneum (2) lung (2)					
*E*. *ortleppi*	BAL (1)						
*E*. *equinus*					liver (1)		
*T*. *martis*	peritoneum (1)						

The number of positive cases per biopsy material is given in brackets.

* diverse material (mostly liver)

§ *Macaca fascilcularis*, *Cercopithecus albogularis*, *Lemur catta*

BAL, bronchoalveolar lavage

Next, we were interested in evaluating whether the PCR test validated in this study could identify *E*. *multilocularis* infections in patients which were tested negative in serology. Since serological diagnostics is more reliable in the case of alveolar echinococcosis [16], we exclusively concentrated on *E*. *multilocularis* infected patients. For 40 of the cases with a positive PCR result that indicated an *E*. *multilocularis* infection (exclusively for biopsy material), we also had carried out serological analysis using a highly sensitive *in house* assay that bases on crude antigen material isolated from *E*. *multilocularis* tissue that had been cultivated in *M*. *unguiculatus* [33]. In previous studies, this test system had shown significantly higher sensitivity than assays systems that based on recombinant antigens [36, 37]. In 35 of the 40 cases identified by PCR, the patients also showed positive serology in the crude antigen ELISA test. In 5 cases, however, we had patients with clear negative (n = 3) or borderline (n = 2) serology that nevertheless gave positive results in the PCR assay. In the respective cases of negative serology, positive PCR was obtained from samples taken from spleen, muscle, and liver. In case of the two borderline serologies, the sample material for PCR derived from extra-hepatic locations; i.e. liver and lung. Overall, these data indicated that PCR analysis of biopsy material, using the settings described above, can successfully complement serology in the diagnosis of echinococcosis.

### PCR evaluation of defined human tissue sections

**Fig 4:**
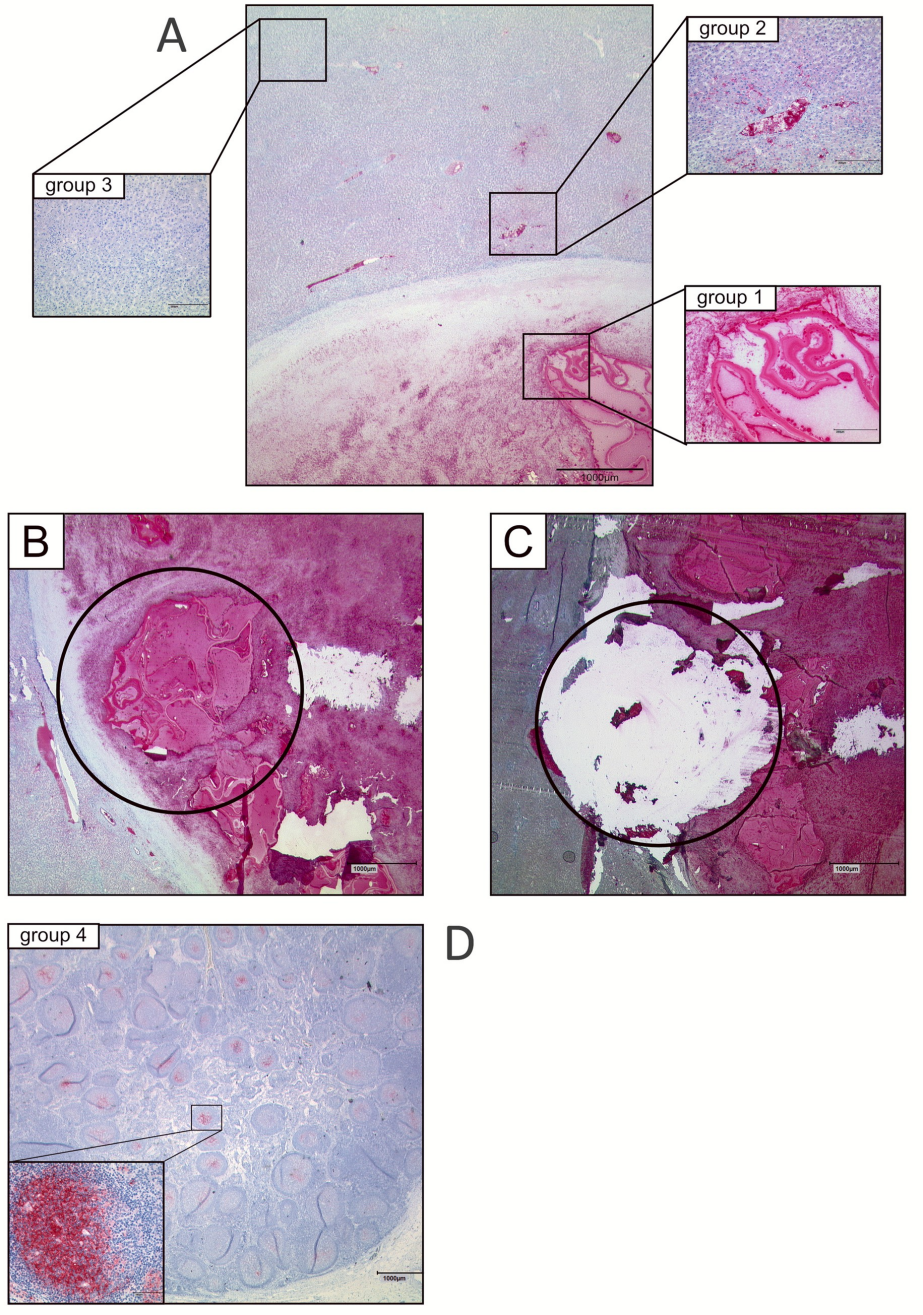
Preparation and localization of samples. A: mAbEm2G11-IHC: Example of different tissue areals of a liver lesion (group 1: tissue areal with laminated layer; group 2: tissue areal with *spems*; group 3: tissue areal without mAbEm2G11-positive material); mAbEm2G11-IHC slide before (B) / after (C) microdissection; D: mAbEm2G11-IHC: lymph node with *spems* (group 4); insert shows higher magnification of a germinal center with *spems*.

After validation of the PCR protocol on isolated parasite DNA, on defined parasite cell numbers, and on biopsies of experimentally and naturally infected hosts, we set out for validation on defined specimen of clinical relevance. In these analyses, we also investigated the nature of the previously reported *spems*, which are parasite structures that contain LL and are located outside of the main lesions in the human host [18]. Since primer-set 1 turned out to be more sensitive for detecting *Echinococcus* DNA in biopsies than primer-set 2 (see above), we decided to carry out all following analyses using primer-set 1 (and PCR protocol A). Overall, 45 pathology samples derived from human tissue of *E*. *multilocularis* infected patients were analyzed. Of these, 20 samples had a conservation age in paraffin of 5 years or lower whereas 25 samples had a conservation time of more than 5 years. As controls, two not infected lymph nodes and two buffer samples were tested (Fig 4).

**Fig 5:**
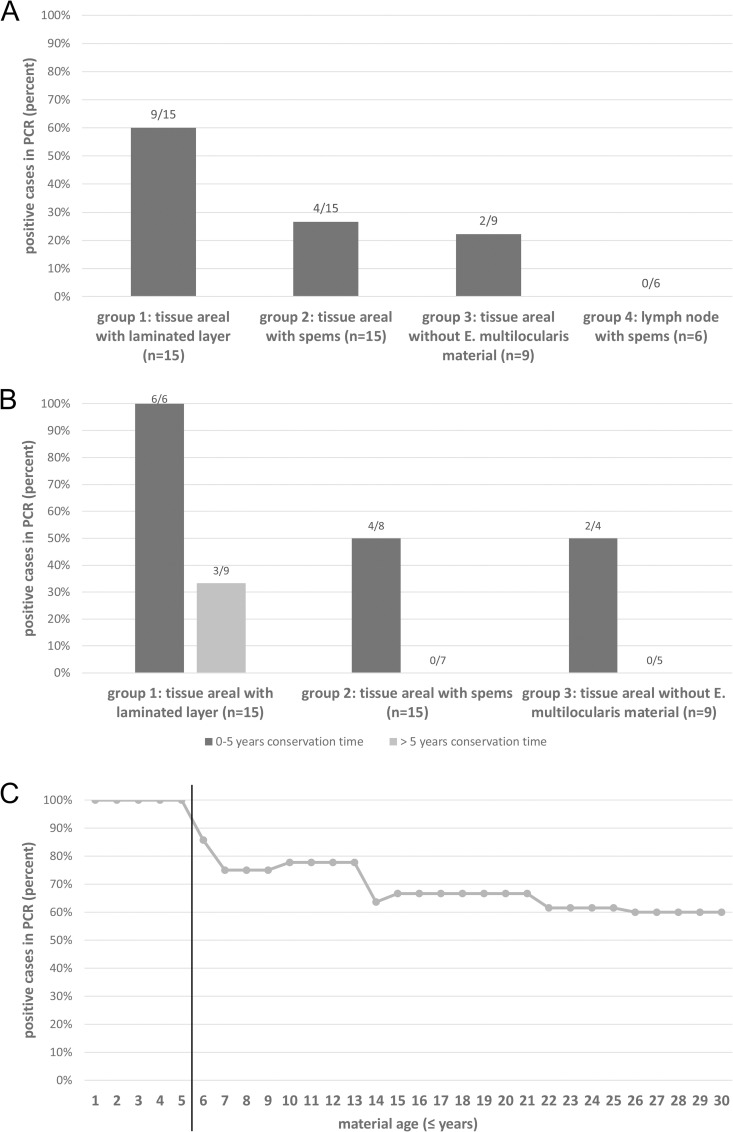
Results of cestode-PCR on FFPE samples of AE patients. A: PCR results for all FFPE samples included in the study, subdivided in the different groups; B: Influence of conservation time on PCR results in group 1, 2 and 3. Results for samples stored 5 years or less are represented in dark grey, those for samples stored longer than 5 years in light gray. C: Time course of PCR-results of group 1; subdivided in samples ≤ 5 years conservation time and > 5 years conservation time.

As expected, 4 of 4 negative control samples were tested negative by PCR. Furthermore, all 6 samples of test group 1 (parasite lesions with LL and germinal layer) with a conservation time of 5 years or lower were tested positive (Fig 5), indicating that the PCR is very well designed to detect *E*. *multilocularis* parasite material in tissue sections (as has already been shown above). In samples of group 1 that had been stored for longer than 5 years, however, only three of nine samples yielded positive results, indicating a negative influence of storage time on the sensitivity of the test. In test group 2 (tissue areal with *spems*), 4 of 8 (50%) samples that had been stored for five or less years tested positive whereas all seven samples of this group with an age of more than five years tested negative (Fig 5). Interestingly, in cases of *spems* with positive test result, the distance to the main lesion was less than 10 mm. In test group 3 (tissue areal without visible *E*. *multilocularis* material), no sample stored for longer than 5 years (n = 5) yielded a positive result whereas in the samples of storage less than 5 years, two of four (50%) yielded a positive result. Again, in these cases the distance to the lesion was less than 10 mm. Finally, in test group 4 (lymph nodes with *spems*) no sample yielded a positive result irrespective of whether they had been stored for less or longer than 5 years (Fig 5). Taken together, these results indicated an influence of the time of paraffin storage of the samples on the reliability of the test. If stored for longer than 5 years, parasite DNA was not amplified from a small fraction of samples that should normally yield positive results. In fresh samples or in samples stored for less than 5 years, the PCR test yielded positive results for all samples, and still detected parasite DNA in lesions up to a distance of 10 mm to active lesions. In lymph nodes with *spems* material, we did not obtain positive signals, indicating that these do not contain *Echinococcus* cellular material. The PCR-results are given in Fig 5 as well as in S1 and S2 Tables.

Since DNA degradation in FFPE samples of longer storage is most probably due to DNA fragmentation [38], the utilization of shorter amplicons up to 200 bp has been suggested as possible improvement [39]. We therefore designed another primer (12S-newR2) which, together with primer 12S-newF should amplify a fragment of 287 nt and tested this combination on isolated parasite cells mixed with 25 mg liver tissue of *M*. *unguiculatus* and against selected FFPE samples. When tested on isolated parasite cells, this primer pair was indeed suitable to reliably detect as little as 10^2^ cells mixed with laboratory host tissue (S3 Fig). However, in 15 FFPE samples of 5 patients which had been stored between 4 and 22 years we obtained the same results as with primer-set 1 and PCR protocol A (S3 Fig). Hence, at least in the case of our samples, the utilization of a shorter amplicon did not yield improved resolution.

Like in the case of patients analyzed at the consultant laboratory, we were interested whether the FFPE cohort contained patients that had negative serology but could be positively tested by PCR. In this setting it only makes sense to analyze patients for which we had samples of group 1 (lesions with laminated layer), which applied to 9 of the 19 patients (S1 Table). In 4 cases (patients number 4, 6, 8, 9) we had both positive results in serology and in PCR. In three cases (patients 1, 10, 11) serology had been positive but PCR negative. However, the material of all these patients had been stored for more than 5 years (S1 Table) which, as we have seen, negatively influences PCR. In two cases (patients 2, 5), however, we could detect the infection by PCR although these patients had a negative serology. These data, again, indicated that PCR analysis is a suitable tool for the diagnosis of echinococcosis in cases of negative or doubtful serology.

## Discussion

During recent years, several PCR based tests for the detection of *Echinococcus* tissue in patient samples had been presented, which all targeted mitochondrial loci such as *nd1* [20], 12S rRNA [21, 22], or *cox1* [40–43]. However, with the exception of two systems [40, 43], all these protocols were directed against *E*. *granulosus* and were tested on paraffin fixed cystic echinococcosis patient material. Furthermore, although some of these investigations tested sensitivities of their assays on isolated parasite DNA (between 5 and 50 pg/μl), none of them has engaged in fully validating their methodology on isolated parasite DNA, on matrix effects of host tissue, on isolated parasite cells in clinically relevant settings, and on a wide variety of clinical samples from human and animal origin. We herein show that a PCR protocol which had previously been used to identify *E*. *multilocularis* DNA in liver samples of 9 patients [35] and a newly established protocol directed against *Echinococcus* 12S rDNA (primer-set 2; PCR protocol B49) both reliably detect parasite DNA at a concentration of 1 pg/μl, which is more sensitive than previously established methods [40]. Using isolated parasite primary cells, which are strongly enriched in germinative (stem) cells, we calculated the detection limit to around 50 parasite cells for primer-set 1 and 500 cells for primer-set 2, either for parasite cells alone or when mixed with natural intermediate host liver tissue (*Meriones*) or isolated human liver cells. Using *in vitro* cultivated metacestode material, we also determined the average cell density to about 0,013 cells/μm^3^ which would account to ca. 13.000 cells in a cube of 100 μm side length. Even if biopsy material of only 500 μm side length would be gathered, this would (with estimated 1,6 x 10^6^ *Echinococcus* cells) be far above the detection limit of the method we validated. Taken together, these data clearly indicate that in principle both PCR protocols we analyzed would be highly efficient to detect parasite DNA in biopsy samples. Even in a distance of 1 cm to full blown lesions, we yielded positive results, which was most probably due to parasite DNA material that had leaked out of the lesion.

Using primer-set 1 as the detection method, we also showed that parasite DNA can reliably be identified in biopsy material from humans and animals. Since the validated PCR protocol routinely combines PCR amplification with DNA sequencing (thus yielding 100% specificity), species identification in doubtful cases (based on imaging or serology) can be carried out within 2 working days. Apart from *E*. *multilocularis*, against which we carried out the technical validation, we also positively detected *E*. *granulosus*, *E*. *ortleppi*, and *E*. *equinus*, indicating that all types of *Echinococus* infections in humans and life-stock can be diagnosed. We also positively identified a very rare case of *T*. *martis* in humans [44] and, although we have not yet tested other taeniid cestodes, we assume that primer-set 1 reliably covers a variety of species of the genera *Echinococcus* and *Taenia*.

As expected, the majority of PCR positive samples was detected in liver and lung, which is also the general majority of samples we had to analyze. However, we also positively identified the parasite in samples from unusual infection sites such as the peritoneum or muscle, or material containing parasite leakage such as sputum or ascites. However, it should be pointed out that in the majority of cases, sputum and ascites led to negative results since it probably does not contain sufficient parasite cells if liver or lung lesions are still intact. Interestingly, in two cases we could identify parasite DNA in lymph node samples. Up to date it is not clear by which means hepatic lesions can disseminate within the human host to form metastases in other organs. It is, however, highly likely that this dissemination involves germinative stem cells of the parasite, which are the only metacestode cells that are mitotically active [6], and which are highly enriched in parasite primary cell preparations that have been analyzed in this study. This indeed points to lymph node infections by *Echinococcus* germinative cells as one possibility of spreading within the host. Further interesting is that we could clearly PCR diagnose AE in 5 cases in which a highly sensitive serological test (the crude antigen test) yielded negative or borderline results. Hence, we propose the validated PCR protocol as a promising complementary approach in the diagnosis of echinococcosis, particularly in cases with doubtful imaging and/or serology results.

Several PCR methods had previously been developed to detect *E*. *granulosus* DNA in paraffin-embedded samples [20–22, 41] and, accordingly, using primer-set 1 we could also reliably distinguish between cystic and alveolar echinococcosis in recently prepared paraffin block samples. To further validate the method on paraffin embedded material, and to address the nature of the previously described *spems* [18], we performed PCR evaluation on a defined cohort of FFPE tissue samples. As expected, the PCR reliably detected *E*. *multilocularis* DNA of tissue samples from the center of the lesion in case the material was not older than five years (100% of samples). However, the detection rate in FFPE material conserved for longer than five years was only 3 of 9 samples. The most likely reason for this is a loss of DNA stability in conserved FFPE material, as has been previously described [38]. It has already been suggested that in such cases the application of shorter amplicons might lead to higher resolution [39] but at least in our case, the utilization of an amplicon of around 200 bp did not yield improved results. This does not exclude, however, that the utilization of alternative primers, shorter amplicons, or specific DNA purification methodology for FFPE material (e.g. the QIAamp DNA FFPE Tissue Kit) might significantly improve PCR amplification. Nevertheless, in the case of material older than 5 years, IHC with the monoclonal antibody Em2G11 is still a feasible alternative method for the detection of the LL, which may be used on formalin fixed material for even more than 60 years (patient 14) [18].

In the natural intermediate host, we had detected *Echinococcus* DNA not only in the lesion but also in a distance of up to 1 cm in adjacent liver tissue. Accordingly, we also detected parasite DNA in liver tissue of human AE cases when taken in close proximity (10 mm) to AE lesions, irrespective of whether they contained *spems* or not. Again, we suppose that this was due to parasite DNA that leaked out of either intact (e.g. by *Echinococcus* extracellular vesicles) [45] or damaged parasite tissue.

*spems* had previously been described as small, *E*. *multilocularis*-derived structures that stain positive for LL-specific antibody mAbEm2G11, that are present in the sinusoids of liver tissue adjacent to necrotic lesions [18], and that are significantly increased in amount surrounding parasite lesions in patients under chemotherapy [28]. Furthermore, lymph nodes of AE patients are frequently affected by *spems* [46, 47]. To clarify whether *spems*, in addition to LL structures, also contain cellular material (including stem cells) of the germinal layer, we performed parasite-specific PCR on several samples that had been taken from a larger distance to the main lesion, or from lymph nodes. However, in none of these cases did we obtain positive results, which strongly indicates that they do not contain cellular material or sufficient amounts of parasite DNA. The likeliest explanation concerning the nature of *spems* is, thus, that these structures are shed off the surface of the LL during parasite growth and, are, subsequently transported away from the lesion into the lymph nodes, possibly as a result of the immune response around the parasite. As a consequence, it is unlikely that spreading of the parasite within the human host, which necessarily requires germinative cells, is mediated by *spems*. This does not exclude, however, that *spems* are not involved in modulating the immune response around the parasite, as has been well described in the case of *E*. *granulosus* [7, 48, 49]. Furthermore, it also does not exclude that occasionally both *spems* and cellular parasite material could occur in lymph nodes of patients.

## Conclusions

We herein present a validated and reliable method for PCR detection of *Echinococcus* (and *Taenia*) DNA in clinical biopsy samples and paraffin embedded tissue that complements current serological and imaging approaches for echinococcosis diagnostics. In combination with IHC methods using mAbEm2G11, specific for the parasite LL, the PCR method validated in this work can even in cases of negative serology and doubtful imaging identify *Echinococcus* infections with minute amounts of biopsy material containing 50–500 parasite cells. We used the PCR detection method on clinical samples that contain *spems* and found that these do not contain parasite cells. *spems* therefore highly likely derive from shedding of the LL during parasite expansion. Immunomodulatory activities of *spems* around parasite lesions and in lymph nodes require further investigation.

## Supporting information

S1 Table PCR-results and serology of AE-patients. (DOCX)

S2 Table Number and tissue origin of different sample groups. (DOCX)

S1 Fig Comparison of primer sets 1 and 2 in detecting *Echinococcus* 12S rDNA from isolated DNA and biopsies. PCR has been performed using as a template *E*. *multilocularis* DNA isolated from protoscoleces (lanes 1,2,5,6,9,10) and from parasite lesions of an experimental host (lanes 3,4,7,8,11,12). Primer sets and PCR protocols were: Primer set 1, PCR protocol A in lanes 1–4; primer set 2 and PCR protocol B30 in lanes 5–8; primer set 2 and PCR protocol B49 in lanes 9–12. M indicates the size marker lane. 1,5% agarose gel stained with ethidium bromide.
(PDF)

S2 Fig Cell counts in *E*. *multilcoularis* metacestode vesicle. *E*. *multilocularis* metacestode vesicles have been grown in vitro and nuclei were stained with propidium iodide essentially as previously described [5, 6]. Shown is a Z-stack microscopic image (above) of germinal layer with nuclei (bar represents 10 mm). Shown below are cell counts per mm^3^ germinal layer in five independ experiments (SD, standard deviation).
(PDF)

S3 Fig Comparison of primer-set 1 and primer-set 3 in detecting *E*. *multilocularis* DNA in experimental and clinical samples. A) Agarose gel showing the results of PCR amplification of primer-set 3 on isolated *E*. *multilocularis* cells mixed with 25 mg liver tissue of mongolian jirds. M, marker lane; 1, no *Echinococcus* cells; 2, 10^2^ cells; 3, 10^3^ cells; 4, 10^4^ cells; 5, 10^5^ cells; 6, 10^6^ cells. Marker fragment sizes are indicated to the left (in bp). B) PCR results for selected FFPE samples. Indicated are the patient number (*as listed in S1 Table), the age of the material, the sample group, and the PCR results for primer-set 1 (PCR protocol A) and primer-set 3 (PCR protocol C). + indicates positive result,—indicates negative result.
(PDF)
