# Supplementary material for: Establishing and evaluation of a polymerase chain reaction for the detection of Echinococcus multilocularis in human tissue
Source: PLoS Negl Trop Dis. 2021 Feb 25;15(2):e0009155. doi: 10.1371/journal.pntd.0009155 (PMC7906421; doi:10.1371/journal.pntd.0009155)
Supplement: S1 Table — (DOCX) [file pntd.0009155.s001.docx]

**Table S1. PCR-results and serology of AE-patients**

| **No.** | **age**  **(years)** | **sex** | **PNM^a^** | **material**  **age (years)** | **sample**  **group^b^** | **tissue** | **PCR** | **ELISA IgG** |
| --- | --- | --- | --- | --- | --- | --- | --- | --- |
| 1 | 70 | female | PXN1M1 | 14 | group 1 | mamma | negative | positive |
|  |  |  |  | 14 | group 1 | heart | negative | positive |
| 2 | 41 | female | P2N0M0 | 15 | group 4 | lymph node | negative | negative |
|  |  |  |  | 15 | group 1 | liver | positive | negative |
|  |  |  |  | 15 | group 2 | liver | negative | negative |
|  |  |  |  | 15 | group 3 | liver | negative | negative |
| 3 | unknown | unknown | n/a | 4 | group 1 | liver | positive | unknown |
|  |  |  |  | 4 | group 2 | liver | positive | unknown |
|  |  |  |  | 4 | group 3 | liver | negative | unknown |
| 4 | 67 | male | PXN1M1 | 26 | group 1 | soft tissue | positive | positive |
|  |  |  |  | 26 | group 1 | soft tissue | negative | positive |
|  |  |  |  | 26 | group 2 | soft tissue | negative | positive |
| 5 | 29 | female | P2N0M0 | 10 | group 4 | lymph node | negative | negative |
|  |  |  |  | 10 | group 1 | liver | positive | negative |
|  |  |  |  | 10 | group 2 | liver | negative | negative |
|  |  |  |  | 10 | group 3 | liver | negative | negative |
|  |  |  |  | 10 | group 2 | liver | negative | negative |
| 6 | 35 | male | P2N0M0 | 5 | group 1 | liver | positive | positive |
|  |  |  |  | 5 | group 2 | liver | negative | positive |
|  |  |  |  | 5 | group 3 | liver | negative | positive |
| 7 | 25 | female | P2N0M0 | 5 | group 4 | lymph node | negative | positive |
| 8 | 39 | female | P4NXMX | 4 | group 4 | lymph node | negative | positive |
|  |  |  |  | 4 | group 1 | liver | positive | positive |
|  |  |  |  | 4 | group 2 | liver | positive | positive |
|  |  |  |  | 4 | group 3 | liver | positive | positive |
| 9 | 24 | male | P4N1M0 | 2 | group 4 | lymph node | negative | positive |
|  |  |  |  | 2 | group 1 | liver | positive | positive |
|  |  |  |  | 2 | group 2 | liver | positive | positive |
|  |  |  |  | 2 | group 3 | liver | positive | positive |
| 10 | 44 | female | P2N0M0 | 22 | group 1 | liver | negative | positive |
|  |  |  |  | 22 | group 2 | liver | negative | positive |
|  |  |  |  | 22 | group 3 | liver | negative | positive |
| 11 | 21 | female | P3N1M0 | 6 | group 4 | lymph node | negative | positive |
|  |  |  |  | 6 | group 1 | liver | negative | positive |
|  |  |  |  | 6 | group 2 | liver | negative | positive |
| 12 | 66 | female | n/a | 14 | group 2 | liver | negative | unknown |
|  |  |  |  | 14 | group 3 | liver | negative | unknown |
| 13 | 56 | female | n/a | 1 | group 2 | cytology | positive | unknown |
|  |  |  |  | 1 | group 1 | cytology | positive | unknown |
| 14 | 28 | male | n/a | 68 | group 1 | brain | negative | unknown |
|  |  |  |  | 68 | group 3 | brain | negative | unknown |
| 15 | 35 | male | PXN1MX | 1 | group 2 | cytology | negative | positive |
| 16 | 78 | male | n/a | 1 | group 2 | liver | negative | unknown |
| 17 | 71 | male | P2N0M0 | 1 | group 1 | liver | positive | negative |
| 18 | 33 | male | P1N0M0 | 1 | group 2 | liver | negative | negative |

^a^PNM (Parasitic mass in the liver, involvement of Neighbouring organs, Metastasis) classified according to Kern et al., 2006; ^b^sample groups: group 1: tissue areal with laminated layer; group 2: tissue areal with spems; group 3: tissue areal without E. multilocularis material; group 4: lymph node with spems; n/a: not available
