# Supplementary material for: Establishing and evaluation of a polymerase chain reaction for the detection of Echinococcus multilocularis in human tissue
Source: PLoS Negl Trop Dis. 2021 Feb 25;15(2):e0009155. doi: 10.1371/journal.pntd.0009155 (PMC7906421; doi:10.1371/journal.pntd.0009155)
Supplement: S2 Table — (DOCX) [file pntd.0009155.s002.docx]

**S2 Table.** **Number and tissue origin of different sample groups**

|  |  | **n** | **tissue origin** | |  |  |  |  |  |
| --- | --- | --- | --- | --- | --- | --- | --- | --- | --- |
|  |  |  | liver | brain | mamma | heart | cytology | soft tissue | lymph node |
| **tissue samples of E. multilocularis infected patient** | | 45 (15 positive PCR/ 30 negative PCR) | 29 (12/17) | 2 (0/2) | 1 (0/1) | 1 (0/1) | 3 (2/1) | 3 (1/2) | 6 (0/6) |
|  | **group 1: tissue areal with laminated layer** | 15 (9/6) | 9 (7/2) | 1 (0/1) | 1 (0/1) | 1 (0/1) | 1 (1/0) | 2 (1/1) | 0 (0/0) |
|  | **group 2: tissue areal with spems** | 15 (4/11) | 12 (3/9) | 0 (0/0) | 0 (0/0) | 0 (0/0) | 2 (1/1) | 1 (0/1) | 0 (0/0) |
|  | **group 3: tissue areal without E. multilocularis material** | 9 (2/7) | 8 (2/6) | 1 (0/1) | 0 (0/0) | 0 (0/0) | 0 (0/0) | 0 (0/0) | 0 (0/0) |
|  | **group 4: lymph node with spems** | 6 (0/6) | 0 (0/0) | 0 (0/0) | 0 (0/0) | 0 (0/0) | 0 (0/0) | 0 (0/0) | 6 (0/6) |
