# Supplementary material for: Establishing and evaluation of a polymerase chain reaction for the detection of Echinococcus multilocularis in human tissue
Source: PLoS Negl Trop Dis. 2021 Feb 25;15(2):e0009155. doi: 10.1371/journal.pntd.0009155 (PMC7906421; doi:10.1371/journal.pntd.0009155)
Supplement: S1 Fig — PCR has been performed using as a template E. multilocularis DNA isolated from protoscoleces (lanes 1,2,5,6,9,10) and from parasite lesions of an experimental host (lanes 3,4,7,8,11,12). Primer sets and PCR protocols were: Primer set 1, PCR protocol A in lanes 1–4; primer set 2 and PCR protocol B30 in lanes 5–8; primer set 2 and PCR protocol B49 in lanes 9–12. M indicates the size marker lane. 1,5% agarose gel stained with ethidium bromide. (PDF) [file pntd.0009155.s003.pdf]

Figure S2

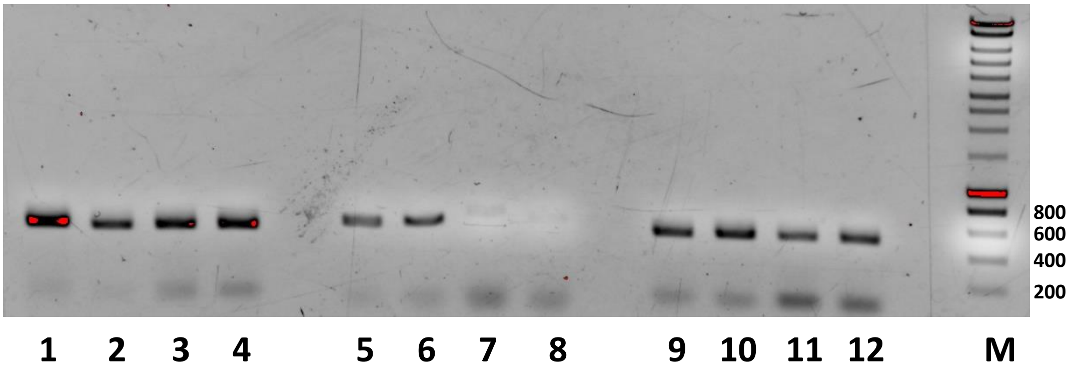

Figure S2: Comparison of primer sets 1 and 2 in detecting *Echinococcus* 12S rDNA from isolated DNA and biopsies. PCR has been performed using as a template *E. multilocularis* DNA isolated from protoscoleces (lanes 1,2,5,6,9,10) and from parasite lesions of an experimental host (lanes 3,4,7,8,11,12). Primer sets and PCR protocols were: Primer set 1, PCR protocol A in lanes 1-4; primer set 2 and PCR protocol B30 in lanes 5-8; primer set 2 and PCR protocol B49 in lanes 9-12. M indicates the size marker lane. Marker band sizes (in bp) are indicated to the right of the marker lane. 1,5% agarose gel stained with ethidium bromide.
