# Supplementary material for: Establishing and evaluation of a polymerase chain reaction for the detection of Echinococcus multilocularis in human tissue
Source: PLoS Negl Trop Dis. 2021 Feb 25;15(2):e0009155. doi: 10.1371/journal.pntd.0009155 (PMC7906421; doi:10.1371/journal.pntd.0009155)
Supplement: S2 Fig — E. multilocularis metacestode vesicles have been grown in vitro and nuclei were stained with propidium iodide essentially as previously described [5, 6]. Shown is a Z-stack microscopic image (above) of germinal layer with nuclei (bar represents 10 mm). Shown below are cell counts per mm3 germinal layer in five independ experiments (SD, standard deviation). (PDF) [file pntd.0009155.s004.pdf]

Figure S3

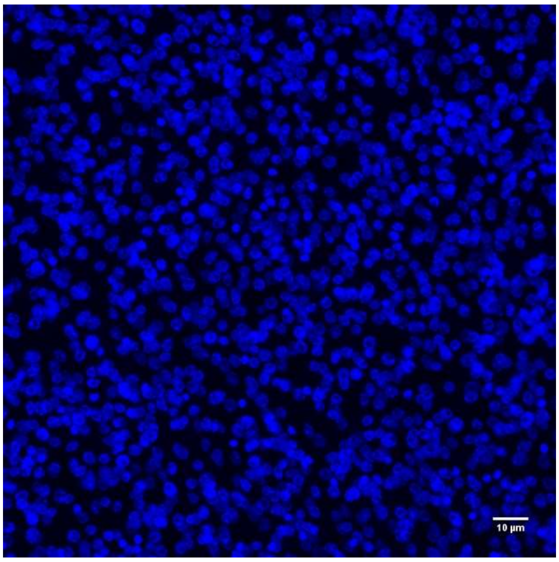

	cells counted	vol (μm3)	cells/μm^3
Vesicle 1	509	384400	1,3 x 10^{-3}
Vesicle 2	800	184400	2,1 x 10^{-3}
Vesicle 3	500	192200	2,6 x 10^{-3}
Vesicle 4	813	624650	1,3 x 10^{-3}
Vesicle 5	1200	624650	1,9 x 10^{-3}
average			1,8 x 10^{-3}
SD			0,5 x 10^{-3}

Fig. S3: Cell counts in *E. multilcoularis* metacestode vesicle. E. multilocularis metacestode vesicles have been grown in vitro and nuclei were stained with propidium iodide essentially as previously described [5,6]. Shown is a Z-stack microscopic image (above) of germinal layer with nuclei (bar represents 10 μm). Shown below are cell counts per μm^3 germinal layer in five independ experiments (SD, standard deviation).
