# Supplementary material for: Establishing and evaluation of a polymerase chain reaction for the detection of Echinococcus multilocularis in human tissue
Source: PLoS Negl Trop Dis. 2021 Feb 25;15(2):e0009155. doi: 10.1371/journal.pntd.0009155 (PMC7906421; doi:10.1371/journal.pntd.0009155)
Supplement: S3 Fig — A) Agarose gel showing the results of PCR amplification of primer-set 3 on isolated E. multilocularis cells mixed with 25 mg liver tissue of mongolian jirds. M, marker lane; 1, no Echinococcus cells; 2, 102 cells; 3, 103 cells; 4, 104 cells; 5, 105 cells; 6, 106 cells. Marker fragment sizes are indicated to the left (in bp). B) PCR results for selected FFPE samples. Indicated are the patient number (*as listed in S1 Table), the age of the material, the sample group, and the PCR results for primer-set 1 (PCR protocol A) and primer-set 3 (PCR protocol C). + indicates positive result,—indicates negative result. (PDF) [file pntd.0009155.s005.pdf]

Figure S5

A

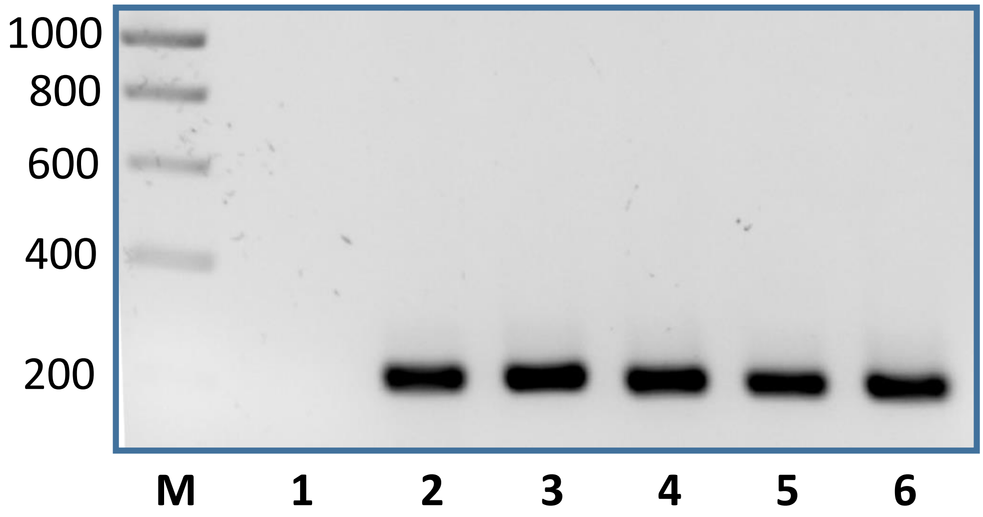

B

patient number*	material age (years)	sample group	Primer-set 1	Primer-set 3
2	15	1	+	+
		2	-	-
		3	-	-
3	4	1	+	+
		2	+	+
		3	-	-
8	4	1	+	+
		2	+	+
		3	+	+
10	22	1	-	-
		2	-	-
		3	-	-
11	6	1	-	-
		2	-	-
		3	-	-

Figure S5: Comparison of primer-set 1 and primer-set 3 in detecting *E. multilocularis* DNA in experimental and clinical samples. A) Agarose gel showing the results of PCR amplification of primer-set 3 on isolated *E. multilocularis* cells mixed with 25 mg liver tissue of mongolian jirds. M, marker lane; 1, no *Echinococcus* cells; 2, 10^2 cells; 3, 10^3 cells; 4, 10^4 cells; 5, 10^5 cells; 6, 10^6 cells. Marker fragment sizes are indicated to the left (in bp). B) PCR results for selected FFPE samples. Indicated are the patient number (*as listed in Table S1), the age of the material, the sample group, and the PCR results for primer-set 1 (PCR protocol A) and primer-set 3 (PCR protocol C). + indicates positive result, - indicates negative result.
